# A Good check on the Bayes factor

**DOI:** 10.3758/s13428-024-02491-4

**Published:** 2024-09-04

**Authors:** Nikola Sekulovski, Maarten Marsman, Eric-Jan Wagenmakers

**Affiliations:** Department of Psychology, University of Amsterdam, Amsterdam, Netherlands

**Keywords:** Weight of evidence, Bayesian hypothesis testing, Turing

## Abstract

Bayes factor hypothesis testing provides a powerful framework for assessing the evidence in favor of competing hypotheses. To obtain Bayes factors, statisticians often require advanced, non-standard tools, making it important to confirm that the methodology is computationally sound. This paper seeks to validate Bayes factor calculations by applying two theorems attributed to Alan Turing and Jack Good. The procedure entails simulating data sets under two hypotheses, calculating Bayes factors, and assessing whether their expected values align with theoretical expectations. We illustrate this method with an ANOVA example and a network psychometrics application, demonstrating its efficacy in detecting calculation errors and confirming the computational correctness of the Bayes factor results. This structured validation approach aims to provide researchers with a tool to enhance the credibility of Bayes factor hypothesis testing, fostering more robust and trustworthy scientific inferences.

## Introduction

The Bayes factor (Kass & Raftery, 1995; Jeffreys, 1935) serves as a valuable tool for testing scientific hypotheses by comparing the relative predictive adequacy of two competing statistical models. In recent decades, there has been a surge in the adoption of Bayes factors as a tool for hypothesis testing (e.g., in psychology, Heck et al., 2023; van de Schoot et al., 2017). This increasing trend towards Bayesian hypothesis testing and model comparison has been catalyzed by a growing critique of traditional frequentist null hypothesis significance testing methods (e.g., Wasserstein and Lazar, 2016; Wagenmakers, 2007; Cohen, 1994; Wagenmakers et al., 2018b; Benjamin et al., 2018; for an early critique see Edwards et al., 1963). In addition, the emergence of user-friendly software packages (e.g., JASP Team, 2023; Morey and Rouder, 2022; Gu et al., 2021) and associated tutorial articles have played a crucial role in making the benefits of the Bayesian framework more accessible to applied researchers (e.g., van Doorn et al., 2021; Rouder et al., 2012; Hoijtink et al., 2019; Marsman and Wagenmakers, 2017; Wagenmakers et al., 2018b; Wagenmakers et al., 2018a). Overall, this upswing in Bayesian methodology has ushered in a new era of statistical analysis, offering researchers valuable alternatives to traditional approaches.

Although Bayes factors have gained popularity in scientific practice, calculating them can be challenging, especially when comparing the relative likelihood of two complex models, such as hierarchical or nonlinear models with a large number of parameters. In such cases, Bayes factors often need to be approximated using various numerical (sampling) techniques such as bridge sampling (Gronau et al., 2017) or path sampling (Zhou et al., 2012); for a general introduction to stochastic sampling in Bayesian inference see Gamerman and Lopes (2006). These techniques often require the user to specify proposal distributions or tune certain parameters within the sampler, which may lead to inaccuracies. There are also state-of-the-art sampling methods designed to obtain joint posterior probabilities over many models; some notable examples of these transdimensional methods are Reversible Jump MCMC (Green, 1995), MCMC with mixtures of mutually singular distributions (Gottardo & Raftery, 2008) and the product space method (Lodewyckx et al., 2011; Carlin & Chib, 1995). These methods, even though very powerful, are quite complex to implement in software and therefore error-prone. Therefore, despite their utility, the use of these numerical techniques can introduce errors, such as the one highlighted by Tsukamura and Okada (2023), who pointed out a common coding error when computing Bayes factors in certain settings in the Stan programming language (Stan Development Team, 2023). Recently, Schad and Vasishth (2024) showed that Bayes factor estimates can be biased in some commonly used factorial designs.

In addition to the potential inaccuracies of existing approac-hes, ongoing research is constantly advancing the methods used to compute Bayes factors; a recent development by Kim and Rockova (2023) introduces a deep learning estimator as an addition to the toolkit of techniques available for computing Bayes factors. While the diversity of computational approaches is crucial, it is important to note that the complexity of these tools can lead to inaccuracies in Bayes factor calculations in applied research contexts. Thus, the development of appropriate controls and checks becomes imperative.

Schad et al. (2022) highlight five key considerations that warrant attention when computing Bayes factors, two of which are (i) the Bayes factor estimates for complex statistical models can be unstable, and (ii) the Bayes factor estimates can be biased. Therefore, Schad et al. (2022) propose a structured approach based on simulation-based calibration, which was originally developed as a method to validate the computational correctness of applied Bayesian inference more generally, and use it to verify the accuracy of Bayes factor calculations (see Talts et al., 2018; Cook et al., 2006; Geweke, 2004). Their method is based on the idea that that the marginal expected posterior model probability is equal to the prior model probability. We provide a more detailed description of the method proposed by Schad et al. (2022) in one of the following sections.

Before proposing another formal Bayes factor check in the spirit of the one by Schad et al. (2022), we would like to mention two other methods that, while not explicitly described as Bayes factor checks, can be used for this purpose. For the first method, suppose a researcher is interested in computing the Bayes factor for the relative adequacy of two complex (possibly non-nested) models, $\mathcal {H}_1$ and $\mathcal {H}_{\text {2}}$, and has already chosen a numerical method implemented in some software for computing $\text {BF}_{12}$. To check that the calculation has been carried out correctly, they can construct nested versions of each of the models by selecting a single parameter and setting it to its maximum likelihood estimate (MLE) value, which would act as a surrogate oracle null model. They can then use the Savage–Dickey density ratio (Dickey & Lientz, 1970; Wagenmakers et al., 2010) to compute $\text {BF}_{\text {ou}}$ – the Bayes factor in favor of the oracle null over the unconstrained model – for both $\mathcal {H}_1$ and $\mathcal {H}_{\text {2}}$. When both models have Savage–Dickey $\text {BF}_{\text {ou}}$’s that match the $\text {BF}_{\text {ou}}$’s obtained from the method under scrutiny, then this gives the researcher reason to believe that $\text {BF}_{12}$ has been computed correctly. A similar approach has been implemented by Gronau et al. (2020) for computing the marginal likelihood in evidence accumulation models, achieved by introducing a Warp-III bridge sampling algorithm. A second method to check the Bayes factor is pragmatic and can be used whenever multiple computational methods are available for a specific application. The idea is that one can use all methods – if they agree, they will mutually reinforce the conclusion and provide evidence that the Bayes factor has been calculated correctly. Furthermore, a Bayes factor can be computed for this agreement. Given that the probability of two correct methods yielding the same outcome is 1, the Bayes factor is calculated as 1 divided by the probability of a chance agreement between two methods, assuming at least one is incorrect. Since the probability of two methods converging on the same wrong value is very small, the Bayes factor provides very strong evidence that both methods are correct.

In this paper, we draw attention to two theorems by Alan Turing and Jack Good (e.g., Good, 1950, 1985, 1994), which they proposed could be used to verify the computation of Bayes factors. We introduce a structured approach to perform this verification, aiming to revive and highlight an idea that, until now, has not received the attention it deserves.

The remainder of this paper is structured as follows. In the next section, we provide an overview of the material in Good (1985), where we discuss the theorems, introduce key concepts, and establish notation. Following this, we present a simple binomial model to illustrate the conditions under which these theorems apply. Next, we outline the workflow for the Bayes factor check tool and offer two numerical examples to demonstrate its application-one employing an ANOVA design and the other utilizing a complex psychometric network model. We conclude the paper by comparing the strengths and limitations of this method, as well as highlighting potential avenues for improvement.

## Theoretical background

### The weight of evidence

Good (1985) points out that the concept of *weight of evidence*, which is used in many areas (e.g., in science, medicine, law, and daily life), is a function of the probabilities of the data under two hypotheses (see also Good, 1950, 1965, 1979, 1994, 1995). Formally, this relation takes the form

$$\begin{aligned} \mathcal {W}(\mathcal {H}_1:\text {data}) = f[p(\text {data} \mid \mathcal {H}_1), p(\text {data} \mid \mathcal {H}_2)], \end{aligned}$$

where $\mathcal {W}(\mathcal {H}_1:\text {data})$ denotes the weight of evidence in favor of the hypothesis $\mathcal {H}_1$ provided by the evidence (data), while $p(\text {data} \mid \mathcal {H}_\cdot )$ denote the probabilities of the data under each of the hypotheses (i.e., what is usually called the marginal likelihood of the data). Good (1985) further points out that this function should be mathematically independent of $p(\mathcal {H}_\cdot )$, known as the prior probability of a hypothesis, but that $p(\mathcal {H}_\cdot \mid \text {data})$ (i.e., the posterior probability) should depend both on the weight of evidence and the prior probability. This relationship can therefore be expressed as

1 $$\begin{aligned} \underbrace{\frac{p(\mathcal {H}_1\mid \text {data})}{p(\mathcal {H}_2 \mid \text {data})}}_{\begin{array}{c} \text {posterior}\\ \text { odds} \end{array}} = \underbrace{\frac{p(\mathcal {H}_1)}{p(\mathcal {H}_2)}}_{\begin{array}{c} \text {prior}\\ \text {odds} \end{array}} \times \underbrace{\frac{p(\text {data} \mid \mathcal {H}_1)}{p(\text {data} \mid \mathcal {H}_2)}}_{\begin{array}{c} \text {Bayes}\\ \text {factor} \ (\text {BF}_{12}) \end{array}}. \end{aligned}$$

Thus, the Bayes factor can be interpreted as the factor by which the initial odds are multiplied to give the final odds, or as the ratio of the posterior odds for $\mathcal {H}_1$ to its prior odds. When $\mathcal {H}_1$ and $\mathcal {H}_2$ are simple (point) hypotheses the Bayes factor is equal to the likelihood ratio (Royall, 2017). Good defined the weight of evidence as the logarithm of the Bayes factor (Good, 1950, 1985, 1994), because it is additive and symmetric (e.g., $\log (\text {BF} = 10) = 2.3$ and $\log (\text {BF} = {1}/{10}) = -2.3$, the average of which is 0). In contrast, the Bayes factor scale is not symmetric – the average of a Bayes factor of 10 and 1/10 is larger than 1. In writing about an appropriate metric for the weight of evidence, Good (1985) draws attention to a counterintuitive theorem about the Bayes factor, and suggested it may be used to check whether a particular procedure computes Bayes factors correctly. The theorem states that *“the expected (Bayes) factor in favor of the false hypothesis is 1”*. Good attributed this paradoxical insight to Alan Turing, whose team at Bletchley Park decrypted German naval messages during World War II (cf. Zabell, 2023).

In the following subsection, we first introduce Turing’s theorem. We then present another related theorem proposed by Good, which shows the relationship between higher-order moments of Bayes factors.

### Moments of the Bayes factor

#### *Theorem 1: The expected (Bayes) factor in favor of the false hypothesis equals 1. – Alan Turing*

##### Proof

Suppose the possible outcomes of an experiment are $E_1, E_2,...,E_M$, where $\mathcal {H}_t$ is the true hypothesis and $\mathcal {H}_f$ is the false hypothesis.^1^ Taking the expectation of the Bayes factor in favor of one of the hypotheses simply means calculating the weighted average of that Bayes factor where the weights are provided by the probability of the evidence given the true hypothesis (i.e., $p(\text {E} \mid \mathcal {H}_t)$). Then the expected Bayes factor in favor of $\mathcal {H}_f$ is given by

$$\begin{aligned} \mathbb {E}[\text {BF}_{ft} \mid \mathcal {H}_t ]\ &= \sum _{i = 1}^{M} \frac{p(\text {E}_i \mid \mathcal {H}_f)}{p(\text {E}_i \mid \mathcal {H}_t)} \times p(\text {E}_i \mid \mathcal {H}_t) \\&= \sum _{i = 1}^{M} p(\text {E}_i \mid \mathcal {H}_f) = 1. \end{aligned}$$

$$\square $$

The theorem states that the expected Bayes factor against the truth is 1, regardless of sample size. For example, consider a binomial experiment with $n = 2$ trials and *k* successes, where $\mathcal {H}_0\text {: } \theta = {1}/{2}$ and $\mathcal {H}_1\text {: } \theta \sim \text {Beta}(\alpha =1\text {, }\beta =1)$. There are three possible outcomes for this experiment, $E_1\text {: } k = 0$, $E_2\text {: } k = 1$, and $E_3\text {: } k = 2$. It follows from the beta-binomial distribution that the probability is the same for each possible outcome under $\mathcal {H}_1$, which in this case is 1/3 $\forall \ E_i$. Under $\mathcal {H}_0$ the probability of $E_1$ and $E_3$ is 1/4 and for $E_2$ is 1/2. Assuming that $\mathcal {H}_1$ is the correct hypotheses we have

$$ \mathbb {E}[\text {BF}_{01} \mid \mathcal {H}_1]\ = 2 \times \frac{{1}/{4}}{{1}/{3}} \times {1}/{3} + \frac{{1}/{2}}{{1}/{3}} \times {1}/{3} = 1. $$

As a Bayes factor of 1 indicates the complete absence of evidence, this theorem is paradoxical; intuition suggests that – especially for large sample sizes – the average Bayes factor against the truth should be much smaller than 1. As mentioned in the previous subsection, unlike the weight of evidence, the Bayes factor is not symmetric. For example, the mean of $\text {BF}_{10} = {1}/{10}$ and $\text {BF}_{10} = 10$ is 5.05 and not 1, whereas the mean of $\log ({1}/{10})$ and $\log (10)$ is 0. This theorem implies that the sampling distribution of the Bayes factor is skewed to the right. Therefore, Good (1985) suggests that the Bayes factor is likely to have a (roughly) log-normal distribution while the weight of evidence has a (roughly) normal distribution (see also, Good, 1994). Finally, Good (1985) shows that the expected weight of evidence in favor of the truth (i.e., $\mathcal {W}(\mathcal {H}_t: \text {data})$) is non-negative and vanishes when the weight of evidence is 0. This again illustrates that the weight of evidence is additive and its expected value is more meaningful than that of the Bayes factor.

Until now, Theorem 1 has been used almost exclusively to establish the universal bound on obtaining misleading evidence (e.g., Royall, 2000; Sanborn and Hills, 2014). The universal bound states that the probability of obtaining a Bayes factor greater than or equal to ${1}/{\alpha }$ in favor of the false hypothesis is less than or equal to some threshold $\alpha$. For example, the probability of obtaining a Bayes factor of 100 in favor of the false hypothesis is less than or equal to $1\%$. This is related to the fact that a Bayes factor *in favor of the false hypothesis* is related to a non-negative test martingale where the expected value of the martingale at any point *t* is 1.^2^ That is, the test martingale measures the evidence against a hypothesis $\mathcal {H}$, and its inverse at some point *t* is a Bayes factor in favor of $\mathcal {H}$ (see e.g., Shafer et al., 2011; Grünwald et al., 2020).^3^ These properties have also been used independently in sequential analysis by Abraham Wald (Wald, 1945). Since the concept of a martingale (Ville, 1939) predates the work of Good and Turing, this suggests that they were not the first to be (at least implicitly) aware of this theorem. However, Jack Good was apparently the first to propose that the theorem may be used to verify the computation of the Bayes factor (Good, 1985, p. 255). This paper implements Good’s idea.

Theorem 1 shows that the first moment of the Bayes factor under the false hypothesis is equal to 1. This is the main result; however, Good (1985) shows that Theorem 1 is a special case of another theorem which shows the equivalence between higher-order moments of Bayes factors; we turn to this theorem next.

#### *Theorem 2: Equivalence of moments for Bayes factors under*$\mathcal {H}_1$*and*$\mathcal {H}_2$*. – Jack Good*

The second theorem generalizes the first and states that

$$ \mathbb {E}[\text {BF}_{12}^k \mid \mathcal {H}_1] = \mathbb {E}[\text {BF}_{12}^{k + 1} \mid \mathcal {H}_2]. $$

##### Proof

The theorem can be expressed as

$$\begin{aligned} \sum _{i = 1}^{M} \left( \frac{p(\text {E}_i \mid \mathcal {H}_1)}{p(\text {E}_i \mid \mathcal {H}_2)} \right) ^{k} \,&\times \; p(\text {E}_i \mid \mathcal {H}_1) = \sum _{i = 1}^{M} \left( \frac{p(\text {E}_i \mid \mathcal {H}_1)}{p(\text {E}_i \mid \mathcal {H}_2)} \right) ^{k + 1} \,\\&\times \; p(\text {E}_i \mid \mathcal {H}_2). \end{aligned}$$

Using the product law of exponents, the right-hand side of the equation above can be rewritten as

$$ \sum _{i = 1}^{M} \left( \frac{p(\text {E}_i \mid \mathcal {H}_1)}{p(\text {E}_i \mid \mathcal {H}_2)} \right) ^{k} \,\times \; \left( \frac{p(\text {E}_i \mid \mathcal {H}_1)}{p(\text {E}_i \mid \mathcal {H}_2)} \right) \,\times \; p(\text {E}_i \mid \mathcal {H}_2), $$

which immediately proves the result.

$$\square $$

This theorem states that the $k^{th}$ moment of the Bayes factor in favor of $\mathcal {H}_1$ about the origin, given that $\mathcal {H}_1$ is true is equal to the $(k+1)^{st}$ moment of the Bayes factor in favor of $\mathcal {H}_1$ given that $\mathcal {H}_2$ is true. Here we refer to the raw moments, that is the moments about the origin and not to the central moments (such as the variance, which is the second moment about the mean). When $k = 0$, this result reduces to that of the first theorem.

Considering the binomial example from earlier with $n = 2$ and hypotheses $\mathcal {H}_0\text {: } \theta = {1}/{2}$ and $\mathcal {H}_1\text {: } \theta \sim \text {Beta}(\alpha = 1\text {, } \beta = 1)$ one can see that

$$\begin{aligned} \mathbb {E}[\text {BF}_{10} \mid \mathcal {H}_1]&= \mathbb {E}[\text {BF}_{10}^2 \mid \mathcal {H}_0] \\&= 2 \times \frac{{1}/{3}}{{1}/{4}} \times {1}/{3} + \frac{{1}/{3}}{{1}/{2}} \times {1}/{3} \\&= 2 \times \left( \frac{{1}/{3}}{{1}/{4}}\right) ^2 \times {1}/{4} + \left( \frac{{1}/{3}}{{1}/{2}}\right) ^2 \times {1}/{2} \\&= 1.11 \end{aligned}$$

## Numerical illustrations

Consider a sequence of *n* coin tosses that forms the basis of a test of the null hypothesis $\mathcal {H}_0\text {: } \theta = {1}/{2}$ against the alternative hypothesis $\mathcal {H}_1\text {: } \theta \sim \text {Uniform}(0,1)$, where $\theta$ represents the probability of the coin landing heads.^4^ Additionally, in the last part of this section, we consider a restricted (directional) hypothesis $\mathcal {H}_{\text {r}}\text {: } \theta > {1}/{2}$. We simulated a total of $m = 100{,}000$ data sets either under $\mathcal {H}_0$, $\mathcal {H}_1$ or $\mathcal {H}_{\text {r}}$ for sample sizes of $n = \{10, 50, 100\}$. For each simulation setting, we averaged the $m = 2, \dots , 100{,}000$ Bayes factors in favor of the wrong hypothesis. The code to reproduce the examples in this paper is publicly available in an OSF repository at https://osf.io/438vy/.

**Fig. 1:**
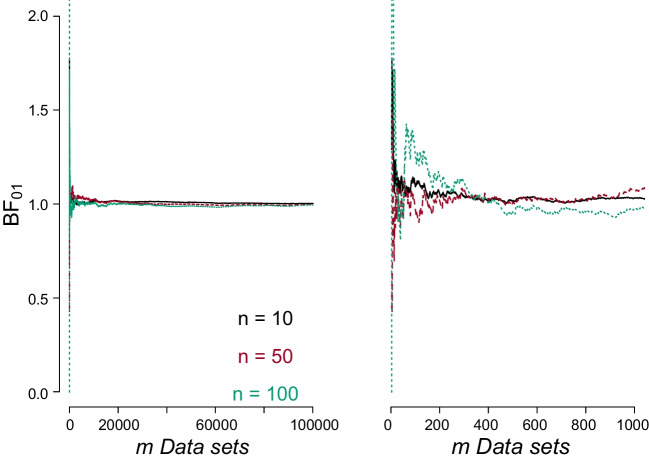
The average Bayes factor in favor of the null hypothesis quickly converges to 1 for synthetic data sets generated under the alternative hypothesis. The figure depicts the average $\text {BF}_{01}$ as a function of the number of synthetic data sets *m* generated under $\mathcal {H}_1$, for $n = 10, 50, 100$; the *black solid line* is for $n = 10$, the *red dashed line* is for $n = 50$, and the *green dotted line* is for $n = 100$. The *left panel* plots the cumulative mean across $m = 100{,}000$ data sets; the *right panel* zooms in on the first $m = 1{,}000$ iterations

**Fig. 2:**
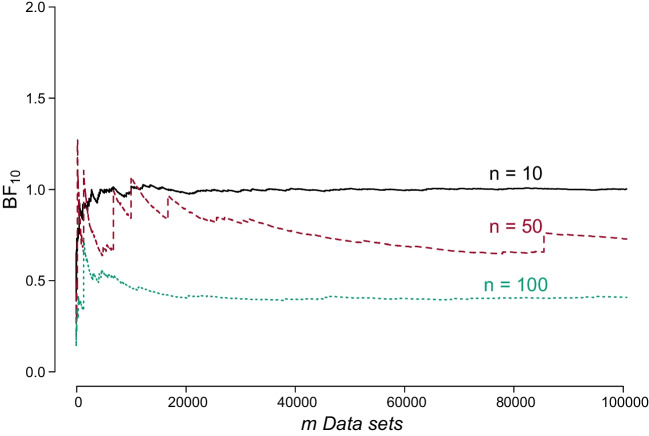
The average Bayes factor in favor of the alternative hypothesis does not converge to 1 as *n* increases for the synthetic data sets generated under the null hypothesis. The figure depicts the average $\text {BF}_{10}$ as a function of the number of synthetic data sets *m* generated under $\mathcal {H}_0$, for $n = 10, 50, 100$; the *black solid line* is for $n = 10$, the *red dashed line* is for $n = 50$, and the *green dotted line* is for $n = 100$

### Illustration of Theorem 1

Figure 1 illustrates the situation where $\mathcal {H}_1$ is true and plots the mean Bayes factor in favor of $\mathcal {H}_0$, that is, the average $\text {BF}_{01}$. For all three values of *n*, the average $\text {BF}_{01}$ quickly stabilizes towards 1. There is a slightly larger instability in the mean for larger sample sizes *n*; however, the results quickly converge as *m* increases.

Figure 2 illustrates the situation where $\mathcal {H}_0$ is true and plots the mean Bayes factor in favor of $\mathcal {H}_1$, that is, the average $\text {BF}_{10}$ calculated for the data sets simulated under $\mathcal {H}_0$. It is immediately evident that for larger sample size *n*, the mean $\text {BF}_{10}$ becomes unstable and moves away from 1. As *m* increases, the average appears to stabilize on values different from 1. This observation suggests that under $\mathcal {H}_0$, with a large sample size, a very large number of iterations would be necessary to obtain a mean $\text {BF}_{10}$ that approaches 1. This phenomenon arises because, under $\mathcal {H}_0$, there exist rare outcomes that produce extreme $\text {BF}_{10}$ values, a situation that does not occur with $\text {BF}_{01}$ when $\mathcal {H}_1$ is the true hypothesis. The chance of encountering these extreme results under $\mathcal {H}_0$, which in turn yields extreme $\text {BF}_{10}$ values, becomes less probable as the sample size *n* increases. Consequently, in this scenario the mean $\text {BF}_{10}$ does not quickly converge to 1. We conclude that the Turing–Good theorems exhibit more robust performance in practice when the true hypothesis is *not* a point null hypothesis (i.e., when the more complicated hypothesis is true).

**Table 1:** First ($\mathbb {E}$) and second ($\mathbb {E}^2$) raw moments of the Bayes factor when $\mathcal {H}_1$ and $\mathcal {H}_0$ are true, for different *n* calculated analytically and from synthetic data. Matching values have the same cell color

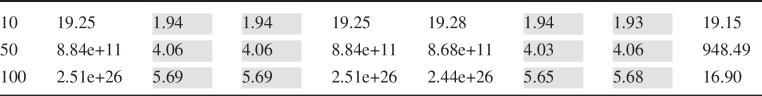	

**Fig. 3:**
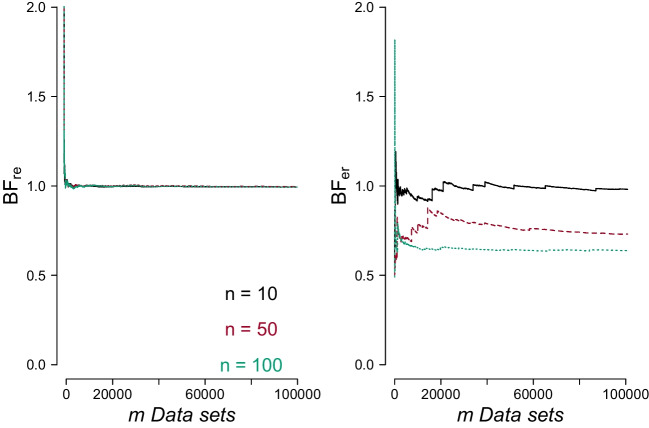
When the encompassing hypothesis is true, the average Bayes factor in favor of the restricted hypothesis rapidly converges to 1, whereas for when the restricted hypothesis is true the average Bayes factor in favor of the encompassing hypothesis does not converge to 1 when the sample size is large. The *left panel* shows the average $\text {BF}_{\text {re}}$ as a function of the number of synthetic data sets *m* generated under $\mathcal {H}_{\text {e}}$, for $n = 10, 50, 100$; the *black solid line* is for $n = 10$, the *red dashed line* is for $n = 50$, and the *green dotted line* is for $n = 100$. The *right panel* shows the average $\text {BF}_{\text {er}}$ as a function of the number of synthetic data sets *m* generated under $\mathcal {H}_{\text {r}}$, for $n = 10, 50, 100$

### Illustration of Theorem 2

To illustrate the second theorem, we compare the first moment of the Bayes factor in favor of the true hypothesis with the second raw moment in favor of the false hypothesis. We first calculated these moments analytically for $n = \{10, 50, 100\}$ with $\mathcal {H}_0\text {: } \theta = {1}/{2}$ and $\mathcal {H}_1\text {: } \theta \sim \text {Uniform}(0,1)$. We then calculated the same moments for the Bayes factors based on the synthetic data. We calculated the second raw moments for the Bayes factors using the following formula:

$$ \mathbb {E}[\text {BF}_{10}^2]=\text {VAR}[\text {BF}_{10}] + \mathbb {E}[\text {BF}_{10}]^2. $$

The results are summarized in Table 1.

The eighth column of Table 1 shows that, on average, the evidence for $\mathcal {H}_0$ increases with the sample size *n*. Comparing the seventh and eighth columns (shaded in gray) confirms that the mean of $\text {BF}_{01}$ when $\mathcal {H}_0$ is true is approximately equal to the second raw moment of $\text {BF}_{01}$ when $\mathcal {H}_1$ is true, regardless of sample size.

**Fig. 4:**
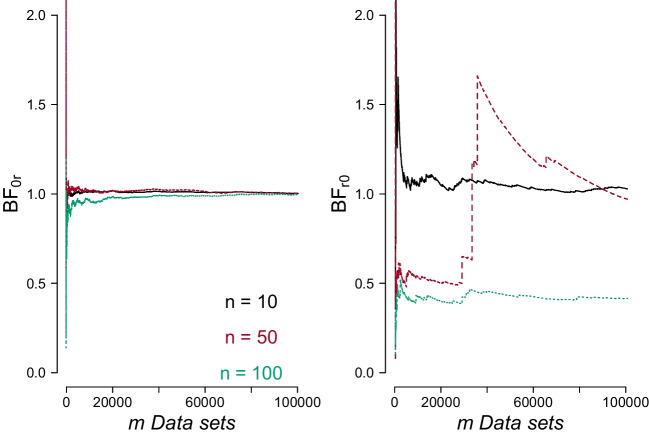
When the restricted hypothesis is true, the Bayes factor in favor of the null hypothesis rapidly converges to 1, whereas when the null hypothesis is true the Bayes factor in favor of the restricted hypothesis does not converge to 1 when the sample size is large. The *left panel* shows the average $\text {BF}_{\text {0r}}$ as a function of the number of synthetic data sets *m* generated under $\mathcal {H}_{\text {r}}$, for $n = 10, 50, 100$; the *black solid line* is for $n = 10$, the *red dashed line* is for $n = 50$, and the *green dotted line* is for $n = 100$. The *right panel* shows the average $\text {BF}_{\text {r0}}$ as a function of the number of synthetic data sets *m* generated under $\mathcal {H}_0$, for $n = 10, 50, 100$

**Table 2:** First ($\mathbb {E}$) and second ($\mathbb {E}^2$) raw moments of the Bayes factor when comparing $\mathcal {H}_{\text {r}}$ with either $\mathcal {H}_{\text {e}}$ or $\mathcal {H}_0$, for different *n* calculated on the synthetic data

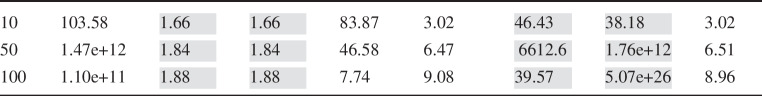	

Matching values have the same cell color

The sixth column of Table 1 shows that the expected evidence in favor of $\mathcal {H}_1$ becomes extreme as *n* increases; contrasting this with the second moment of $\text {BF}_{10}$ when $\mathcal {H}_0$ shows that the values are equal for $n = 10$, but as *n* increases these values diverge. These instabilities are due to the same reasons highlighted in the previous subsection. Note, however, that the theorems still hold in this situation, and for a very large number of iterations *m* the moments are expected to eventually converge. This is supported by the analytical solutions presented in columns 2 through 6. However, the results computed from the synthetic data suggest that in practice, when dealing with a point null hypothesis, one should compute the first moment from the data generated under $\mathcal {H}_0$ and compare it with the second raw moment computed from the data generated under $\mathcal {H}_1$.

It is also possible to compare, for example, the second and third raw moments. In the results from the simulation, the second raw moments of $\text {BF}_{01}$ for the data sets generated under $\mathcal {H}_0$ are 4.28, 19, and 37.32, for $n = 10, 50$, and 100, respectively. And the third raw moments of $\text {BF}_{01}$ for the data sets generated under $\mathcal {H}_1$ are 4.3, 18.8 and 37.1. These results illustrate that the second theorem holds for higher-order moments in general.

### Directional hypotheses

In this subsection, we examine how the Bayes factor behaves when one of the hypotheses under consideration is a directional (i.e., inequality constrained or restricted) hypothesis. Hypotheses that consist of a combination of inequality and equality constraints among the parameters are known as informative hypotheses (Hoijtink, 2011). Informative hypotheses allow researchers to express their substantive theory and expectations and have become popular in recent years; therefore, it is important to also consider how inequality constrained hypotheses perform under the two theorems.

We make use of the restricted hypothesis $\mathcal {H}_{\text {r}}: \theta > {1}/{2}$, which we specify as $\mathcal {H}_{\text {r}}: \theta \sim \text {Uniform}(0.5, 1)$. This is equivalent to setting a truncated Beta distribution from 0.5 to 1 for the probability $\theta$. We then compare $\mathcal {H}_{\text {r}}$ with the alternative hypothesis ($\mathcal {H}_1$) and the null hypothesis ($\mathcal {H}_0$) from the previous subsections. In line with previous literature (e.g., Klugkist et al., 2005), we rename the alternative hypothesis ($\mathcal {H}_1$) to the encompassing hypothesis and denote it as $\mathcal {H}_{\text {e}}$, as both $\mathcal {H}_0$ and $\mathcal {H}_{\text {r}}$ are nested under this encompassing hypothesis.

Figure 3 illustrates the situation of comparing $\mathcal {H}_{\text {e}}$ and $\mathcal {H}_{\text {r}}$. In the left plot, the average $\text {BF}_{\text {re}}$ when $\mathcal {H}_{\text {e}}$ is the true hypothesis quickly stabilizes towards 1 for all three sample size values. Note also that the initial fluctuations are all greater than 1; this is because half of the outcomes expected under $\mathcal {H}_{\text {e}}$ are also plausible under $\mathcal {H}_{\text {r}}$. The right panel of Fig. 3 illustrates the reverse situation, where $\mathcal {H}_{\text {r}}$ is the true hypothesis. As can be seen, the Bayes factor now does not quickly converge to 1 for larger sample sizes, because under $\mathcal {H}_{\text {r}}$, outcomes that produce large $\text {BF}_{\text {er}}$’s are highly improbable; similar to the case when considering $\text {BF}_{10}$ when $\mathcal {H}_0$ is true (cf. Figure 2).

Figure 4 illustrates the situation of comparing $\mathcal {H}_0$ with $\mathcal {H}_{\text {r}}$. In the left panel, the average $\text {BF}_{0r}$ when $\mathcal {H}_{\text {r}}$ is the true hypothesis approaches 1 for all three sample size values; note, however, that for $n = 100$ it takes a considerable number of iterations for the average $\text {BF}_{\text {0r}}$ to converge to 1. The right panel of Fig. 4 illustrates the situation when $\mathcal {H}_0$ is the true hypothesis; as was the case in Fig. 2, when the point (null) hypothesis is the true hypothesis, for a finite number of iterations, the average Bayes factor in favor of the false hypothesis does not converge to 1 as the sample size increases. Again, this is due to the fact that under $\mathcal {H}_0$ very few outcomes produce large $\text {BF}_{\text {r0}}$.

Examining the third and fourth columns of Table 2, we see that the second raw moment of $\text {BF}_{\text {re}}$ when $\mathcal {H}_{\text {e}}$ is true is equal to the mean of $\text {BF}_{\text {re}}$ when $\mathcal {H}_{\text {r}}$ is true. A similar observation can be made when comparing the sixth and ninth columns. This illustrates that the second theorem also holds for inequality-constrained hypotheses. However, if we compare the mean of $\text {BF}_{\text {er}}$ when $\mathcal {H}_{\text {e}}$ is true with the second moment of $\text {BF}_{\text {er}}$ when $\mathcal {H}_0$ is true, we observe that these values diverge, especially as the sample size increases. The same divergence occurs when we compare the mean of $\text {BF}_{12}$ when $\mathcal {H}_1$ is true with the second moment of $\text {BF}_{12}$ when $\mathcal {H}_0$ is true (cf. Table 1).

These results illustrate that both theorems are applicable to directional hypotheses and can be used as a general method for checking Bayes factors. Furthermore, generalizing from all the examples, the first theorem shows more robust performance when the more general (encompassing) hypothesis is true. For the second theorem, the (more) specific hypothesis should be set to true, and the average Bayes factor in favor of the more specific hypothesis should be compared with the second moment of the Bayes factor in favor of the more specific hypothesis when the more general hypothesis is true.

### An exception to the rule

In the philosophy of science, a *universal generalization* is a hypothesis stating that a parameter or characteristic is true for the entire population without exceptions (e.g., all ravens are black). So for the binomial example, this would be equivalent to $\mathcal {H}_0\text {: } \theta = 1$. The two theorems do not hold in this situation, since they require that the true hypothesis (in this case $\mathcal {H}_0$) must assign a non-zero prior mass to all events that are considered plausible under the false hypothesis. In other words, both hypotheses must assign non-zero mass to the same sample space.

## A formal approach for checking the Bayes factor calculation

In their method for checking the calculation of the Bayes factor, Schad et al. (2022) recommend simulating multiple data sets from statistical models (with predefined prior model probabilities) and then obtaining Bayes factors and posterior model probabilities using the same method that is to be used to calculate the Bayes factor(s) on the empirical data. This method represents a structured approach based on simulation-based calibration (Geweke, 2004; Cook et al., 2006). The idea is based on the fact that the expected posterior model probability should equal the prior model probability (see e.g., Skyrms, 1997; Goldstein, 1983; Huttegger, 2017). Therefore, if the average posterior model probability across the simulated data sets is equal to the prior model probability, then the calculation of the Bayes factor (and the posterior model probability) should be considered accurate.

**Fig. 5:**
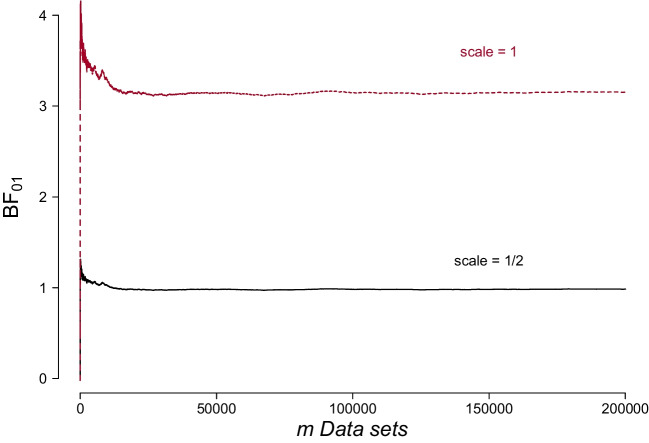
For the correctly specified calculation the average $\text {BF}_{01}$ rapidly converges to 1, whereas for the misspecified calculation, it does not. The figure depicts average $\text {BF}_{01}$ calculated for the data generated under $\mathcal {H}_1$ as a function of the number of synthetic data sets *m*. The Bayes factor is calculated using two different values for the scale of the scaled inverse chi-squared distribution

In this paper, we follow the approach by Schad et al. (2022) and propose a new method for checking the Bayes factor, based on Turing and Good’s theorems described in the previous sections. The check (steps 1-4) assumes that if the calculation of the Bayes factor is executed correctly and if all the assumptions are met, then its expected value in favor of the wrong hypothesis should be (approximately) equal to 1. Additionally, it is possible to extend this check by comparing higher-order moments (steps 5-6). After collecting the data and selecting the appropriate analysis, the proposed methodology can be summarized as follows:

1. Specify two rival models; since the prior can be seen as an integral part of the model (e.g., Vanpaemel, 2010; Vanpaemel and Lee, 2012), this step includes the assignment of prior distributions to the model parameters.
2. Calculate the Bayes factor based on the observed data using the computational methodology of interest.
3.
  1. Select one of the models to generate simulated data from – we strongly recommend this to be the more complex model; in nested models, one should therefore simulate from the alternative hypothesis and not from the null hypothesis.
  2. Sample data from the prior predictive distribution. This could, for example, be done by selecting a parameter (vector) from the joint prior distribution and use this to generate a synthetic data set of the same length as the observed data (although it could be any length in principle).
  3. Compute the Bayes factor in favor of the false hypothesis over the true hypothesis for the synthetic data set, using the same computational technique used for the observed data (step 2).
  4. Repeat steps b-c *m* times, yielding *m* Bayes factors in favor of the false hypothesis.
4. Calculate the average Bayes factor in favor of the false hypothesis across the *m* Bayes factors obtained in the previous step. If this mean value is close to 1 for a sufficiently large number of simulations *m*, this provides strong evidence that the Bayes factor calculation has been executed correctly. Then one can confidently report the value obtained in step 2.
5. Additionally, simulate data as described in step 3, but this time set the other hypothesis under consideration (e.g., $\mathcal {H}_0$) to true. Calculate the Bayes factor in favor of the true hypothesis. Repeat this step *m* times and calculate the average Bayes factor in favor of the true hypothesis.
6. Compare the mean Bayes factor from step 5 with the second moment of the Bayes factor in favor of the wrong hypothesis based on the data generated in step 3. If these two values are approximately equal, this provides additional evidence that the Bayes factor calculation was performed correctly.

This step-by-step approach helps validate the Bayes factor calculations and ensures that the results obtained are reliable. More specifically, if the Bayes factor calculation is done correctly, we should be confident that there were no issues with the calculation of the Bayes factor. In the following two subsections, we illustrate these steps with two concrete examples.

Note that the purpose of the following examples-one using a simple Bayes factor for an intervention effect in an ANOVA design, and another using a transdimensional Bayes factor for the inclusion of an edge in a graphical model-is to demonstrate how to perform the proposed check. A comprehensive review of the performance of various software packages in calculating Bayes factors is beyond the scope of this paper.

### Example 1: A Bayes factor test for an intervention effect in one-way ANOVA

Consider a one-way ANOVA model where the standard alternative hypothesis ($\mathcal {H}_1$), which states that not all means between the 3 groups are equal, is tested against the null hypothesis ($\mathcal {H}_0$), which states that the means are equal. The model can be expressed as

$$\begin{aligned} y_i = \alpha + x_i \beta + \epsilon _i, \end{aligned}$$

where $y_i$ is the value of the dependent variable for participant *i*, $\alpha$ is the intercept, $x_i$ is the factor variable denoting the group membership, $\beta$ is the parameter representing the effect of the experimental manipulation, and $\epsilon _i$ is the residual term normally distributed around 0 with variance $\sigma ^2$. To calculate the Bayes factor on the empirical data one can use the default settings in the R package BayesFactor (Morey & Rouder, 2022). The function anovaBF assigns Jeffreys priors to the intercept and residual variance, and a normal prior to the main effect

$$\begin{aligned} \beta \sim \mathcal {N}(0, g), \end{aligned}$$

where *g* is given an independent scaled inverse-chi-squared hyperprior with 1 degree of freedom. The interested reader is referred to Rouder et al. (2012) for the details of the prior specifications. We now illustrate how the check can be performed for the current example.

Suppose we have collected data from 150 participants (50 participants in each of the 3 groups) and we wish to test $\mathcal {H}_1$ versus $\mathcal {H}_0$. We simulate $m = 200{,}000$ data sets under $\mathcal {H}_1$ by sampling the parameter $\beta$ from its prior distribution, employing the same default specification as used in the package (i.e., applying a scaled inverse-chi-squared hyperprior for *g* with a scale of 1/2 and Jeffreys priors on $\alpha$ and $\sigma ^2$ with a value of $\sigma ^2 = 0.5$). Additionally, we generate *m* datasets under $\mathcal {H}_0$ by setting $\beta = 0$. In both cases, we calculate the Bayes factors using the default settings as described above. To illustrate what happens when the Bayes factor calculation is misspecified, we re-calculate the Bayes factor for the data generated under $\mathcal {H}_1$ by altering the default value for the scale of the inverse chi-squared distribution. Specifically, we change the scale from medium to ultrawide, corresponding to values of 1/2 and 1, respectively. For the Bayes factors calculated on the data sets where $\mathcal {H}_1$ is true, approximately 0.28% of the Bayes factors calculations failed due to computational difficulties.

Figure 5 depicts the cumulative mean for $\text {BF}_{01}$ when $\mathcal {H}_1$ is true. Notably, for the Bayes factors calculated using the default settings of the package, which precisely mirror how the data was generated, the average $\text {BF}_{01}$ rapidly converges to 1. However, when there is a discrepancy between the data and the Bayes factor calculation, which for the purpose of this example was achieved by altering the scale of the inverse chi-squared hyperprior from 1/2 to 1, we notice that the average Bayes factor deviates significantly from 1. It eventually stabilizes at a value of approximately 3.16, illustrating the sensitivity of the Bayes factor when its calculation is misspecified.

For the second set of synthetic data generated under $\mathcal {H}_0$, we calculate the average $\text {BF}_{01}$, which yields a value of 8.18, which we can compare with the second raw moment of $\text {BF}_{01}$ from the data sets where the alternative hypothesis is true, which yields a value of 8.15. This result provides additional proof that the calculation of the Bayes factor was done correctly.

### Example 2: A Bayes factor test for conditional independence in a Markov random field model

Network psychometrics is a relatively new subdiscipline in which psychological constructs (e.g., intelligence, mental disorders) are conceptualized as complex systems of behavioral and cognitive factors (Marsman & Rhemtulla, 2022; Borsboom & Cramer, 2013). Psychometric network analysis is then used to infer the structure of such systems from multivariate psychological data (Borsboom et al., 2021). These analyses use graphical models known as Markov Random Fields (MRFs, Kindermann and Snell, 1980; Rozanov, 1982) in which psychological variables assume the role of the network nodes. The edges of the network express the direct influence of one variable on another given the remaining network variables, that is, that they are *conditionally dependent*, and the absence of an edge implies that the two variables are *conditionally independent* (Lauritzen, 2004). The Bayesian approach to analyzing these graphical models (Mohammadi & Wit, 2015; Marsman et al., 2015; Marsman, 2022; Marsman et al., 2023; Williams, 2021; Williams & Mulder, 2020) allows researchers to quantify the evidence in the data for the presence or absence of edges, and thus to formally test for conditional (in)dependence (see Sekulovski et al., 2024, for an overview of three Bayesian methods for testing conditional independence).

Sekulovski et al. (2024) discuss two types of Bayes factor tests for conditional independence. In one test, the predictive success of a particular network structure with the relationship of interest is compared against the same network structure with the relationship of interest removed. One problem with testing for conditional independence in this way is that even for relatively small networks, there are many possible structures to consider, and as Sekulovski et al. (2024) have shown, Bayes factor tests for conditional independence can be highly sensitive to the choice of that network structure. In the second Bayes factor test, we use Bayesian model averaging (BMA, Hoeting et al., 1999; Hinne et al., 2020) and contrast the predictive success of *all* structures with the relationship of interest against the predictive success of *all* structures without that relationship. This is known as the inclusion Bayes factor (Marsman, 2022; Marsman et al., 2023). Sekulovski et al. (2024) showed that the inclusion Bayes factor is robust to variations in the structures underlying the rest of the network. However, the BMA methods for psychometric network analysis required to estimate the inclusion Bayes factor are much more complex and thus more prone to the computational problems identified above. For an accessible introduction to BMA with a specific example on network models, see Hinne et al. (2020) and for an accessible introduction to BMA analysis of psychometric network models, see Huth et al. (2023) and Sekulovski et al. (2024).

**Fig. 6:**
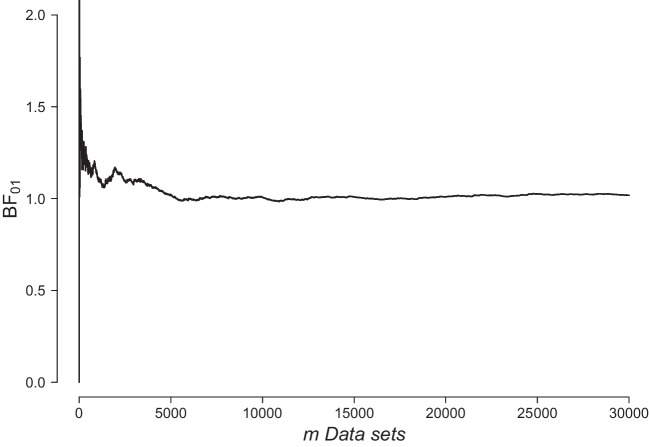
The average $\text {BF}_{01}$ converges to 1. The figure depicts the average inclusion $\text {BF}_{01}$ calculated for the data generated under $\mathcal {H}_1$ as a function of the number of synthetic data sets *m*

In this paper, we scrutinize the Bayesian edge selection method developed by Marsman et al. (2023) for analyzing MRF models for binary and ordinal data, and which can be used to estimate the inclusion Bayes factor. This method, implemented in the R package bgms (Marsman et al., 2023), stipulates a discrete spike and slab prior distribution on the edge weights of the MRF, and models the inclusion and exclusion of pairwise relations in the model with an edge indicator ($\gamma$), which when present designates the corresponding edge weight a diffuse prior and when absent sets it to 0. That is, for a single edge weight $\theta _{ij}$, between variables *i* and *j*, the prior distribution can be expressed as

$$ p(\theta _{ij} \mid \gamma _{ij}) = (1-\gamma _{ij})\, f_\text {spike}(\theta _{ij}) + \gamma _{ij}\ \, f_\text {slab}(\theta _{ij}). $$

The transdimensional Markov chain Monte Carlo method proposed by Gottardo and Raftery (2008) is used to simulate from the multivariate posterior distribution of the MRF’s parameters and edge indicators. The output of this approach can be used to compute the inclusion Bayes factor which is defined as

$$ \underbrace{\frac{p(\text {data} \mid \gamma _{ij} = 1)}{p(\text {data} \mid \gamma _{ij} = 0)}}_ {\begin{array}{c} \text {Inclusion}\\ \text {Bayes factor}\\ (\text {BF}_{10}) \end{array}} = \underbrace{\frac{p(\gamma _{ij} = 1\mid \text {data})}{p(\gamma _{ij} = 0 \mid \text {data})}}_{\begin{array}{c} \text {Posterior}\\ \text {inclusion odds} \end{array}} \bigg / \underbrace{\frac{p(\gamma _{ij} = 1)}{p(\gamma _{ij} = 0)}}_{\begin{array}{c} \text {Prior}\\ \text {inclusion odds} \end{array}}. $$

Since the inclusion Bayes factor is an extension of the classical Bayes factor presented in Eq. 1 and involves a much more complex calculation, we wish to verify that its computation is performed correctly using the newly proposed methodology. Therefore, we simulated $m = 30{,}000$ datasets with $p = 5$ binary variables and $N = 500$ observations each. We focus on testing whether the first two variables are conditionally independent, that is, we compare $\mathcal {H}_0\text {: } \theta _{12} = 0$ with $\mathcal {H}_1\text {: } \theta _{12} \ne 0$. For the case where $\mathcal {H}_1$ is true, we simulated data where all ten possible edges have an edge weight value of $\theta _{ij} = 0.5$. Additionally, for the case where $\mathcal {H}_0$ is true, we simulated a second set of data by setting the focal edge weight parameter $\theta _{12}$ to 0 and leaving the values of the nine remaining edge weights unchanged. We estimated the graphical model for each simulated data set using the R package bgms. We used a unit information prior for $f_{slab}$; a Dirac measure at 0 for $f_{spike}$, and an independent Bernoulli distribution for each $\gamma _{ij}$ with a prior inclusion probability of 1/2 (see Sekulovski et al., 2024, for a detailed analysis of the prior distributions for these models). Under this prior specification, the prior inclusion odds are equal to 1. In cases where the posterior inclusion probability was equal to 1, we obtained undefined values for the inclusion Bayes factor (i.e., 1/0). For the data sets where $\mathcal {H}_1$ was true, there were 9,345 Bayes factors with undefined values (31%), and for the data sets where $\mathcal {H}_0$ was true, there were 53 undefined values (0.2%). To work around this problem, we set all undefined values to 1 + the highest observed finite value of the inclusion Bayes factor.

Figure 6 shows the cumulative mean of the inclusion $\text {BF}_{01}$ when $\mathcal {H}_1$ is true (i.e., there is an edge between variables 1 and 2). As the number of simulations increases, the mean inclusion $\text {BF}_{01}$ stabilizes around 1 (1.01 at the last iteration), indicating that the inclusion Bayes factor obtained with this approach was computed correctly. In addition, we computed the mean $\text {BF}_{01}$ when $\mathcal {H}_0$ is true, which was 11.5, and compared it to the second moment of $\text {BF}_{01}$ when $\mathcal {H}_1$ is true, which was 9.96. These values are not equal. However, we suspect that the reason for this is twofold: first, the sample size *N* in each of the simulated data sets was quite large, and second, since the calculation of this Bayes factor is more involved, it probably takes many more iterations *m* to be sure that the moments are equal. Estimating these models takes much more time than estimating other more standard statistical models, so it was not computationally feasible to do more than $m = 30{,}000$ repetitions under each of the hypotheses. In addition, we must consider the sampling variability of the simulated data sets. In other words, due to variability, not all of the simulated data sets will show support for the hypothesis under which they were simulated, further reducing the number of “effective” data sets. These reasons also justify the choice to recode the undefined inclusion Bayes factor values as we did, rather than omitting them altogether.

## Discussion

This paper presents a structured approach to checking the accuracy of Bayes factor calculations based on the theorems of Turing and Good. The approach provides researchers with a general and practical method for confirming that their Bayes factor results are reliable. Application to two concrete examples demonstrated the effectiveness of this approach in verifying the correctness of Bayes factor calculations. In particular, if the method of calculating the Bayes factor is consistent with the data generation process, the mean Bayes factor in favor of the false hypothesis converges to approximately 1, in accordance with the first theorem. Furthermore, comparing the first and second moments of the Bayes factors under different hypotheses provides additional evidence for correct calculations. However, as we have seen in the second example when dealing with more complex models, the second theorem requires many more iterations. Due to the variability of the second moment, one can only be sure that the second theorem approximately holds for a finite number of simulations. Therefore, we recommend that researchers focus primarily on the first theorem and perform the additional check based on the second theorem whenever practically possible. This would also make the check less computationally expensive since it would only require simulating data under one of the hypotheses.

Finally, we have demonstrated that for practical applications of the first theorem, it is best to simulate under the more general hypothesis and take the average Bayes factor in favor of the more specific hypothesis. For the second theorem, the optimal approach can be summarized as follows. First, compute the mean Bayes factor in favor of the more specific hypothesis for data where that hypothesis is true. Second, compare this to the second raw moment in favor of the more specific hypothesis computed on data simulated under the more general hypothesis.

### Limitations & Possible extensions

While the proposed approach provides a practical way to validate Bayes factor calculations, it is not without limitations. In cases with large sample sizes, or when dealing with highly complex models, the convergence of the values for the higher-order moments may require a significant number of iterations. In such cases, as we have seen, the second moments may not match very closely. In situations where Bayes factors are used for comparing highly complex models, different methods of checking their calculation might be more appropriate, such as the method proposed by Schad et al. (2022).

However, for certain Bayes factors, particularly those based on Bayesian model averaging (BMA), such as the inclusion Bayes factor for including an edge in a graphical model or a predictor in linear regression, the method proposed in this paper can be straightforwardly applied to verify these calculations. This is because the other two methods are more suitable for checking classical (i.e., non-BMA) Bayes factors, which compare two competing statistical models (see, Sekulovski et al., 2024, for a discussion of the difference between these two Bayes factors)

One of the reviewers of the paper suggested that the check proposed in this paper could be incorporated as an additional step within the approach proposed by Schad et al. (2022). This would mean that at the start of the simulation exercise, we would have to (a) assign prior probabilities to *two* competing models and then randomly select one of those models, (b) simulate synthetic data under the sampled model, (c) compute the Bayes factor and the posterior model probability, and then repeat these steps *m* times. Then, step 4 would be split into two, where we filter out the data sets generated by only one of the models, and filter out the associated Bayes factors. For each resulting set of Bayes factors, we would compute the mean in favor of the false hypothesis, where we expect both means to be approximately equal to one.

### Conclusion

Providing a structured and systematic way to evaluate Bayes factor calculations helps to increase the credibility and rigor of Bayesian hypothesis testing in applied research. The proposed methods serve as a valuable tool for researchers working with Bayes factors, providing a means to validate their results and ensure the robustness of their statistical inferences. We encourage researchers to consider this approach when using Bayes factors in their analyses, thereby fostering greater confidence in the validity of their conclusions.

## Data Availability

The data and materials for all simulation examples are available at the OSF repository https://osf.io/438vy/.

## Footnotes

1 We use *E* and ‘data’ interchangeably.

2 A martingale is a sequence of random variables where the conditional expected value of the next value, given all prior values, equals the current value. For instance, consider a coin flip game where a player starts with 100 euros, winning one euro for heads and losing one euro for tails; in this scenario, the expected amount of money after each flip remains equal to the player’s current amount. Consequently, regardless of the number of flips, the expected future value is always equal to the present value, exemplifying the martingale property.

3 It should be noted that the marginal Bayes factor (i.e., a Bayes factor not conditioned on the false hypothesis) is not a martingale.

4 Note that the specification of $\mathcal {H}_0$ and $\mathcal {H}_1$ is the same as in the binomial example from the previous section with $n = 2$.
